# Interactive XAI for personalized and trusted healthcare: need of the hour

**DOI:** 10.1097/JS9.0000000000001643

**Published:** 2024-05-23

**Authors:** Harshita Patel, Aryan Chopra, Dharmendra Singh Rajput, Darli Myint Aung

**Affiliations:** a School of Computer Science Engineering and Information Systems, Vellore Institute of Technology, Vellore, Tamil Nadu, India; b University of Computer Studies (Taunggyi), Myanmar (Burma)

*Dear Editor,*

The groundbreaking research paper by Sheng Li illuminates the profound impact of artificial intelligence (AI) technologies like ChatGPT on the field of surgery^1^. This work focuses on the potential role of ChatGPT in modern surgery, highlighting its significance as AI continues to permeate healthcare. A pressing challenge arises – the opaqueness of AI models hinders patient trust, collaborative decision-making, and personalized care delivery. A recent study by Tonekaboni *et al*.^2^ highlighted how the lack of explainability in AI-driven medical devices leads to reduced treatment adherence and poorer outcomes. This proposal advocates for the implementation of interactive Explainable AI (XAI) as a scientifically grounded solution to these challenges.

The crux of the matter lies in the scientific obstacles faced by opaque ‘black-box’ AI models in healthcare. Firstly, the inability to understand AI reasoning can erode patient confidence, adversely impacting adherence and outcomes^3^. Secondly, the lack of transparency impedes shared decision-making between patients, providers, and AI systems^4^. Thirdly, without insights into model behavior, tailoring care plans to individual needs becomes arduous^5^. However, interactive XAI interfaces allow users to query the AI system, receiving dynamic explanations tailored to their needs – a stark contrast to traditional opaque models.

This interactive XAI approach holds significant scientific potential. On the one hand, opaque AI decisions can undermine patient trust and adherence; on the other hand, interactive explanations demystify AI decisions, fostering patient understanding and trust – a pivotal factor linked to better treatment outcomes^6^. Furthermore, while the lack of transparency hinders collaboration, interactive XAI promotes effective communication between the stakeholders by exposing the AI’s rationale, bolstering shared decision-making^7^. Additionally, user interactions provide valuable feedback to refine AI models, enabling personalized care plans adaptable to evolving patient needs^8^ – a sharp contrast to traditional models’ limited personalization capabilities.

Implementing interactive XAI could yield transformative scientific outcomes. Improved patient trust and adherence are expected, substantiated by studies showing XAI increases trust in AI systems^9^. Moreover, enhanced shared decision-making and collaborative care align with research emphasizing such models’ benefits^10^. Advances in personalized medicine through dynamic, patient-centric AI model refinement are anticipated. Crucially, this endeavor contributes to the ethical and robust development of AI, addressing potential biases and privacy concerns^11^ – a critical consideration often overlooked in the traditional deployment of AI models.

While research on XAI in healthcare is growing, the current landscape exhibits limitations too. Existing approaches predominantly offer post-hoc explanations or static visualizations, limiting real-time interaction and personalization potential. Furthermore, continuous learning in healthcare AI often lacks robust feedback mechanisms to facilitate model adaptation based on user inputs^12^ – a stark contrast to the proposed interactive XAI framework.

To unlock interactive XAI’s full potential, this proposal recommends developing dynamic, user-centric XAI interfaces tailored to healthcare contexts, enabling intuitive querying and comprehensible explanations. Establishing frameworks for continuous learning and model refinement using user feedback, while ensuring data privacy and security is paramount. Conducting mixed-methods research to evaluate how interactive XAI impacts patient trust, treatment adherence, and care team collaboration is crucial. Formulating ethical guidelines governing the responsible development and deployment of interactive XAI and mitigating risks like algorithmic bias is a necessity. Finally, implementing pilot studies with validated outcome measures to assess interactive XAI’s real-world efficacy in improving healthcare experiences and outcomes is imperative.

By pursuing this multifaceted scientific agenda, the healthcare ecosystem can harness interactive XAI’s potential, fostering trust, personalizing care delivery, and propelling the evolution of human-centered AI. In contrast to the shortcomings of traditional opaque models, interactive XAI promises a future where AI-driven healthcare is transparent, collaborative, and tailored to individual patient needs – a paradigm shift toward ethical, trustworthy, and impactful AI integration in healthcare.

## Ethical approval

Not applicable.

## Consent

Not applicable.

## Source of funding

Not applicable.

## Author contribution

H.P.: conceptualization, investigation, writing–original draft, and writing–review and editing; A.C.: data curation and writing–original draft; D.S.R.: investigation, validation, and writing–review and editing; D.M.A.: validation and writing–review and editing: All authors critically reviewed and approved the final version of the manuscript.

## Conflicts of interest disclosure

All authors report no conflicts of interest relevant to this article.

## Research registration unique identifying number (UIN)

Not applicable.

## Guarantor

Harshita Patel, School of Computer Science Engineering and Information Systems, Vellore Institute of Technology, Vellore, Tamil Nadu, India, E-mail: harshita.patel@vit.ac.in.

## Data availability statement

The data in this correspondence article are not sensitive in nature and are accessible in the public domain. The data are therefore available and not of a confidential nature.

## Provenance and peer review

Not commissioned, internally peer-reviewed.
